# Hidden Toxicity in Neonatal Intensive Care Units: Phthalate Exposure in Very Low Birth Weight Infants

**DOI:** 10.4274/jcrpe.3027

**Published:** 2016-09-01

**Authors:** Atalay Demirel, Asuman Çoban, Şükran Yıldırım, Canan Doğan, Rukiye Sancı, Zeynep İnce

**Affiliations:** 1 İstanbul University İstanbul Faculty of Medicine, Department of Pediatrics, Division of Neonatology, İstanbul, Turkey; 2 TÜBİTAK Marmara Research Center, Food Institute, İstanbul, Turkey

**Keywords:** newborn, Preterm, phthalate, exposure, neonatal intensive care units

## Abstract

**Objective:**

To determine exposure to endocrine-disrupting phthalates in preterm infants in neonatal intensive care units (NICU).

**Methods:**

Urine samples (n=151) from 36 preterm infants (<32 weeks of gestation and/or <1500 g of birth weight) were collected on the first 3 days of admission to the NICU and biweekly thereafter. Diethylhexyl phthalate contents of indwelling medical devices used in various procedures and the concentrations of phthalate metabolites in the urine samples were analyzed. The relationships between urinary excretion, exposure intensity, postnatal age and birth weight were examined.

**Results:**

The mean gestational age and mean birth weight of the study infants were 28.9±1.5 weeks and 1024±262 g, respectively. Diethylhexyl phthalate was detected in umbilical catheters, endotracheal tubes, nasogastric tubes, and nasal cannula. Monoethylhydroxyhexyl phthalate (MEHHP) was the most frequently detected metabolite (81.4%); its concentration increased during the first 4 weeks and then started to decrease but never disappeared. Patients who did not need indwelling catheters (except nasogastric tubes) after 2 weeks were classified as group 1 and those who continued to have indwelling catheters as group 2. Although not of statistical significance, MEHHP levels decreased in group 1 but continued to stay high in group 2 (in the 4th week, group 1: 65.9 ng/mL and group 2: 255.3 ng/mL). Levels of MEHHP in the first urinary samples were significantly higher in infants with a birth weight <1000 g (<1000 g: 63.2±93.8 ng/mL, ≥1000 g: 10.9±22.9 ng/mL, p=0.001).

**Conclusion:**

Phthalate metabolites were detected even in the first urine samples of very low birth weight newborns. Phthalate levels were higher in the first weeks of intensive invasive procedures and in preterm infants with a birth weight less than 1000 g. MEHHP was the most frequently detected metabolite and could be a suitable biomarker for the detection of phthalate exposure in preterm infants.

**WHAT IS ALREADY KNOWN ON THIS TOPIC?**

Phthalates are endocrine disruptors and normally should not be present in humans.

**WHAT THIS STUDY ADDS?**

High amounts of phthalate metabolites were detected in the urine samples of neonatal intensive care unit patients and exposure was associated with the intensity and duration of invasive medical procedures.

## INTRODUCTION

Phthalates, which are used as plasticizers, increase the flexibility and durability of polyvinyl chloride (PVC) products. Diethylhexyl phthalate (DEHP) is the most commonly used plasticizer for PVC. Soft PVC can consist of a high percentage of DEHP (up to 40%) (1). Annual phthalate consumption is more than 1 million tons in Western Europe and over 3 million tons globally (2,3). Because of its widespread use, exposure of humans is virtually unavoidable. DEHP is a major component of household products, clothing, packaging, and medical devices. As DEHP is not chemically bound to plastic materials, it can be leached to the environment or directly into the body fluids, thus exposing humans via ingestion, inhalation, dermal absorption, or intravenous route.

Preterm neonates are frequently exposed to numerous medical devices in neonatal intensive care units (NICU). DEHP is a major plasticizer for medical products such as blood and total parenteral nutrition bags, feeding tubes, umbilical catheters, peripherally inserted central catheters, oxygen masks, and endotracheal tubes. The rate of DEHP release depends on the DEHP content of the plastic material, lipophilic nature and flow rate of the solution in contact with the PVC tubing, temperature during use, and storage period (4). In 2005, National Toxicology Program’s Center for the Evaluation of the Risks to Human Reproduction (CERHR) suggested that DEHP exposure of infants in intensive care units may be 2-3 orders of magnitude higher than that of the general adult population and that the level of DEHP exposure may approach the lowest observed adverse effect levels in animal studies (14-23 mg/kg/day) (5).

DEHP is rapidly hydrolyzed to its monoester, monoethylhexyl phthalate (MEHP), which is a minor urinary metabolite of DEHP. MEHP is oxidized to other metabolites such as monoethylhydroxyhexyl phthalate (MEHHP) and monoethyloxohexyl phthalate (MEOHP) which are excreted in the urine in quantities several times greater than MEHP. Thus, these metabolites may be more sensitive biomarkers of DEHP exposure than MEHP. After metabolization, phthalates are rapidly excreted in the urine (90%) and feces (10%), may cross the placenta, and can be detected in breast milk and amniotic fluid (6,7,8).

Phthalates have endocrine-disrupting properties. High-level exposure to phthalates may cause fetal death, malformations, cancer, liver and kidney injury, and reproductive abnormalities in animals (9,10,11). Acute toxicity of phthalates in humans is probably low, but chronic exposure may have reproductive and developmental toxic effects. Fetal and neonatal periods are very sensitive periods for the effects of endocrine disrupters. Exposure to environmental toxic chemicals may increase the incidence of reproductive deficits. Intensive therapeutic interventions and impaired ability to excrete phthalates may explain the susceptibility of preterm infants to high DEHP exposure.

There is limited data on the exposure to DEHP for preterm infants in the NICU. Most of the published data are from animal studies and adult exposure (9,10,11,12,13). However, because of their high exposure rates to DEHP in the NICU and their limited excretion capacity, preterm infants are the most at-risk population (14,15,16,17,18). In this prospective study, we studied the levels of DEHP metabolites in preterm infants who underwent intensive therapeutic medical interventions. This is the first study showing the sequential changes in the levels of DEHP metabolites in preterm infants during their stay in the NICU.

## METHODS

Preterm infants with a gestational age of less than 32 weeks and/or a birth weight of less than 1500 g who stayed in the NICU of the Neonatology Section of the Department of Pediatrics of the İstanbul Faculty of Medicine for at least 2 weeks were enrolled in the study. Infants with major congenital anomalies were excluded. Urine samples were collected from all preterm infants at admission or in their first 3 days in the NICU and every 2 weeks until discharge. A total of 151 urine samples from 36 preterm infants were collected. To determine the effect of exposure intensity and length of exposure on the excretion of phthalate metabolites, two groups of patients were defined: the first group had only a feeding tube as an intervention after two weeks of life, whereas the second group had ongoing interventions using various medical devices (umbilical catheter, nasal cannula, endotracheal tube).

The institutional review board of İstanbul University İstanbul Medical Faculty approved the study protocol. Informed consent was obtained from the parents.

A glass tube was used to collect the urine samples. The tubes were placed around the orifice of the urethral meatus, supported by the diaper, and at least 1 mL of urine was recovered. Urine samples were labelled and frozen immediately at -20 ˚C until analyzed. After all the urine samples were collected, they were sent on dry ice to TÜBİTAK (The Scientific and Technological Research Council of Turkey) for analysis.

Urine samples were analyzed for DEHP and its 3 metabolites, MEHP, MEHHP, and MEOHP. Standard DEHP, MEHP, MEOHP, MEHHP, and MEOHP-^13^C4 solutions were procured from LGC and Cambridge Isotopes Laboratories INC. Acetonitrile and other chemicals (HPLC grade) were purchased from Merck (Darmstadt, Germany). Stock solutions of standards (MEHP, DEHP, MEOHP, and MEHHP) and internal standard (MEOHP-^13^C4) were prepared in acetonitrile [adapted from Kato et al (19) 2004]. Urinary phthalate metabolites were determined by liquid chromatography Tandem Gold quadruple mass spectrometry electrospray ionization (ESI)-liquid chromatography-mass spectrometry (LC-MS)/MS. Cerex system 48, pressure processor vacuum manifold equipment were used for extraction of phthalate analytes prior to LC-MS/MS analysis.

The analytical methods for measuring phthalate metabolites in urine were adapted from Blount et al (12) 2000, Kato et al (20) 2003 and Silva et al (21) 2003. For the free metabolite analysis (non glucuronidated), analytes (1 mL) were spiked with MEOHP-^13^C4 and the phthalate metabolites were extracted from the matrix by solid phase extraction (Oasis HLB, Waters, Milford, MA). The analytes were chromatographically separated by liquid chromatography on a phenyl column (Betasil 5 µm, 50 mmx3 mm) water (acetic acid 0.1%): acetonitrile (acetic acid 0.1%) gradient and analysed by tandem mass spectrometry using electrospray ionization.

All analytes were calibrated linearly with eight standard analyte solutions and six repetitives between 1 ng/mL to 100 ng/mL. The limits of detection were calculated 3S_0_, where S_0_ is the standard deviation value as the concentration approaches zero. S_0_ was determined from the replicate analysis of low-level standards. The relative standard deviations were found below 15%.

All of the samples, blanks, and standards were processed identically using equipment software. Each ion of interest in the chromatogram was automatically selected and integrated. The urinary concentrations were reported in nanograms per milliliter of urine.

DEHP concentrations of unused plastic devices were also determined by gas chromatography and mass spectroscopy (Clarus gas chromatography, Perkin Elmer, Shelton, USA and Clarus 600 T mass spectroscopy, Perkin Elmer, Shelton, USA) and were reported in mg per 0.5 g of that plastic material.

### Statistical Analysis

Statistical Package for Social Sciences (SPSS) 15.0 was used for statistical analysis. For descriptive methods (minimum, maximum, and median) of the two groups, Mann-Whitney U test was used. Friedman test and Wilcoxon rank test were performed for correlations of medians in more than two time periods. Correlation coefficients were calculated by Pearson’s test. The p-value and confidence interval were accepted as <0.05 and 95%, respectively.

## RESULTS

A total of 36 preterm infants (15 boys, 21 girls) with a mean gestational age of 28.9±1.5 weeks (25-31 weeks) and a mean birth weight of 1024±262 g (585-1560 g) were enrolled. The gestational age of 11 patients (31%) was less than 28 weeks, 18 patients (50%) had a birth weight of less than 1000 g, and 9 patients (25%) were small for gestational age. Prolonged rupture of membranes (n=10, 28%) and preeclampsia (n=7, 19%) were the most common pregnancy complications. The majority of the patients (n=31, 86%) were born by cesarean section. Twenty seven patients (75%) had respiratory distress syndrome.

Urinary levels of MEOHP and MEHHP, which are the oxidized metabolites of DEHP, were higher than those of DEHP and MEHP and were also the most commonly detected urinary metabolites (Table 1). As all the metabolites were statistically significantly correlated with each other, the most commonly detected metabolite MEHHP was used as the biomarker of phthalate exposure in our study.

Endotracheal tubes, feeding tubes, nasal cannulas, umbilical catheters, peripherally inserted central catheters, and peripheral lines were the most commonly and long-term used plastic medical devices in our NICU. Phthalates were detected in unused nasal cannulas, feeding tubes, endotracheal tubes, and umbilical catheters. Nasal cannulas had the highest phthalate content (201.7 mg/0.5 g). Phthalate contents of feeding tubes, endotracheal tubes, and umbilical catheters were 71 mg/0.5 g, 29.1 mg/0.5 g, and 3.8 mg/0.5 g, respectively. Peripherally inserted central catheters and peripheral lines had no detectable phthalate content.

**Figure 1:**
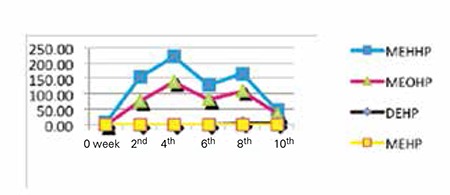
Median levels of phthalate metabolites (ng/mL). DEHP: Diethylhexyl phthalate, MEHP: monoethylhexyl phthalate, MEOHP: monoethyloxohexyl phthalate, MEHHP: monoethylhydroxyhexyl phthalate

The median levels of DEHP, MEHP, MEOHP, and MEHHP are shown in Figure 1. MEHHP levels were higher in the first 2 to 4 weeks of intensive medical intervention and then started to decrease when this period of intensive exposure to plastic materials also decreased, although a small surge has been seen in the eighth week. Only 22 urine samples (14.4% of total urine samples) were collected after the eighth week of the study and MEHHP levels in some of these samples were extremely higher than those in the general study population, probably accounting for the small surge seen in the eighth week. Hence, we used the first six-week levels of MEHHP in the correlations.

**Figure 2:**
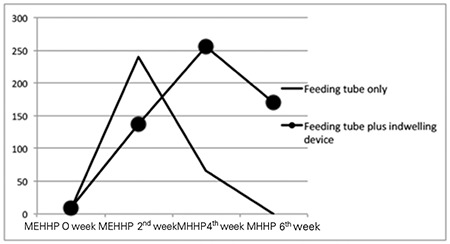
Comparison of median levels of monoethylhydroxyhexyl phthalate (ng/mL) according to exposure intensity and length of exposure. MEHHP: monoethylhydroxyhexyl phthalate

The median urinary levels of MEHHP according to exposure intensity and length of exposure are shown in Table 2 and Figure 2. Although there was no statistically significant difference between the groups, urinary MEHHP levels started to decrease in the first group (no further intervention) but stayed high in the second group (ongoing intervention) with high exposure intensity, extending through the sixth week of life.

When the urinary excretion of MEHHP levels were compared according to birth weight category, the levels in the first urinary samples of extremely low birth weight infants were significantly higher than those of infants with a birth weight of ≥1000 g (p=0.001) although the intensity of interventions were similar between the groups (Table 3).

## DISCUSSION

Phthalate exposure is widespread and unavoidable in NICU. Various medical devices, like feeding tubes, endotracheal tubes and umbilical catheters, contain phthalates as plasticizers. DEHP is the most commonly used phthalate derivative. While the major source of human exposure is ingestion of contaminated food or exposure via the dermal route, in NICU, the newborn infants are exposed to very high doses of phthalates during medical procedures such as mechanical ventilation, parenteral nutrition, or blood transfusion (14). In adults, approximately 80-90% of urinary metabolites of phthalates are conjugated to glucuronic acid and easily excreted, but in newborns and preterm infants, this conjugation pathway is immature (15). Furthermore, renal clearance is reduced due to a low glomerular filtration rate, and this may also increase the toxicity risk of phthalates. In the present study, we prospectively and sequentially studied the excretion of phthalate metabolites in the urine samples of preterm infants during their stay in our level III NICU.

Most of the previous studies have been performed on animals and adults. There are only a few studies in newborn infants and most of them are cross-sectional with a limited number of patients (16,17,18). The first study was reported by Calafat et al (16) who collected 41 urine samples from 6 preterm patients and showed increased DEHP metabolites (MEHP, MEOHP, and MEHHP) in urine samples confirming that newborns who underwent intensive invasive procedures in NICU were exposed to higher concentrations of DEHP than the general population. Green et al (17) showed in 54 newborns in NICUs that intensive use of DEHP-containing medical devices resulted in higher exposure to DEHP as reflected by increased MEHP in their urine samples. In a follow-up report, the same group of researchers investigated the urinary excretion of two additional metabolites of DEHP (MEOHP and MEHHP) in the same group of newborns and showed that inclusion of these metabolites in the analysis strengthened the association between the intensity of product use and exposure to DEHP (18). In our study, we investigated the excretion of DEHP metabolites in a group of high risk neonates sequentially, reporting not only the association with the intensity of exposure to DEHP containing products but also the duration of exposure and birthweight category of the patients.

The presence of DEHP in urinary samples may not always indicate exposure as this substance is a natural environmental contaminant. If plastic urine bags containing DEHP are used in collecting the specimens, this may result in overestimation of phthalate levels. To overcome this handicap, either non-DEHP containing materials such as cotton balls or glass tubes should be used in urine collection or secondary metabolites of DEHP (such as MEOHP or MEHHP) should be measured. We preferred to use glass tubes in urine collections and also measurement of secondary metabolites (MEOHP and MEHHP) in addition to DEHP. Blount et al (12) demonstrated that instead of the mother compound, measurement of phthalate monoesters (MEHP, MEOHP, and MEHHP) may prevent misdiagnosis of exposure. DEHP is rapidly hydrolyzed to MEHP, which may also be formed by a biotic processes such as hydrolysis, oxidation, and photolysis. MEHP is then oxidized to MEOHP and MEHHP. These secondary oxidized metabolites are not susceptible to contamination and are excreted in higher amounts than DEHP and MEHP. Calafat et al (16) reported that urinary concentrations of oxidized metabolites were higher and the concentrations of MEOHP and MEHHP were highly correlated. Due to contamination risks and low frequency of detection of DEHP and MEHP in urine samples, the use of MEOHP and MEHHP as biomarkers of phthalate exposure may be more appropriate. In our study, we showed that the urinary concentrations of MEOHP and MEHHP were highly correlated and that their concentrations were 18.4 and 24.5 times higher than that of MEHP, respectively. In addition to high concentrations, the high detection rate of MEHHP (81.4%) makes it a more suitable biomarker of phthalate exposure.

In order to determine exposure intensity, we measured the phthalate contents of unused medical devices in our NICU. Nasal cannulae had the highest amount of DEHP, followed by feeding tubes, endotracheal tubes, and umbilical catheters. Leakage of DEHP from these devices has been shown in previous studies. Takatori et al (22) simulated neonatal exposure to DEHP and MEHP from enteral nutrition products. They showed a 10-fold increase in the amount of phthalates in enteral nutrition fluid after transfer to PVC-containing enteral feeding bags and catheters. Chiellini et al (23) determined the phthalate contents of used and unused endotracheal tubes reporting the correlation between the leaching of phthalates and the duration of intubation in newborns. Latini and Avery (24) showed DEHP leakage and color change in endotracheal tubes after use in high risk newborns. They examined spectrocolorimetric changes in endotracheal tubes and determined significant color changes signifying in vivo degradation of DEHP-containing devices.

To our knowledge, sequential analysis of phthalate excretion in preterm infants during the course of their NICU stay has not been studied before. We showed that urinary concentrations of MEHHP and MEOHP increased in the first 4 weeks of life when intensive invasive procedures were performed. After this period, levels of DEHP metabolites started to decrease but did not disappear, indicating the persistence of exposure. MEHHP levels decreased in patients who had only feeding tubes after the second week of life, but stayed high in patients who continued to have additional invasive procedures. This result indicates the association between the intensity of exposure and excretion of DEHP metabolites as has been shown in other studies with the additional finding that duration of exposure is also important (17,18).

Concentration of phthalate metabolites may increase with immaturity. Zhang et al (25) showed that MEHP levels in cord blood, meconium and maternal serum samples of low birth weight infants were higher than those of normal birth weight infants. In two other studies, urinary levels of MEOHP and MEHHP in 6 extremely low birth weight infants were found to be approximately 10 times higher than those of 62 older children and adult patients (13,16). In our study we did not compare the levels of DEHP metabolites of the study group with those of term babies, but made a subgroup analysis of those babies with a birthweight less than 1000 g and ≥1000 g. MEHHP levels in the first urine samples of babies <1000 g were significantly higher than those ≥1000 g, although there was no significant difference in the intensity of invasive procedures between the groups. We speculated that this difference may be related to the level of immaturity or other undetermined antenatal exposure. We started to collect urine in the first 72 hours of admission to NICU. So increased levels of MEHHP in the first urine samples may be much more related with immaturity of our patients than maternal exposure. This is the limitation of our study. Future studies investigating maternal exposure, amniotic fluid and cord blood levels of DEHP metabolites in extremely low birth weight infants are needed to clarify this issue.

In conclusion, phthalates are endocrine-disruptors and normally should not be present in humans. There are only a few studies investigating DEHP exposure in preterm babies. The results of our study show that high amounts of oxidized metabolites of DEHP in the urine samples of NICU patients can be detected and that this exposure is associated with the intensity and duration of invasive medical procedures. We showed that MEHHP was a more suitable biomarker of DEHP exposure. We do not know the long-term effects of this exposure during a very sensitive period of life, and we believe this is an issue which should be investigated in further studies. In the meantime, DEHP-free medical devices should be used for the patient population such as preterm infants which may be sensitive to the toxicity of phthalates.

## Ethics

Ethics Committee Approval: The institutional review board of İstanbul University İstanbul Medical Faculty approved the study protocol (2010/716-208, 20.10.2010), Informed Consent: Informed consent was obtained from the parents.

Peer-review: Externally peer-reviewed.

## Figures and Tables

**Table 1:**
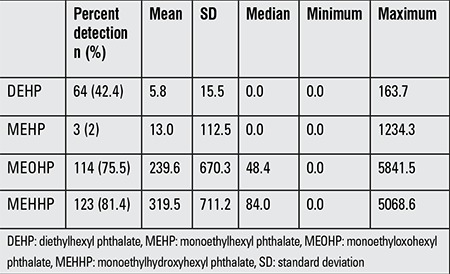
Detection rates and levels of phthalate metabolites (ng/mL) in urine samples

**Table 2:**
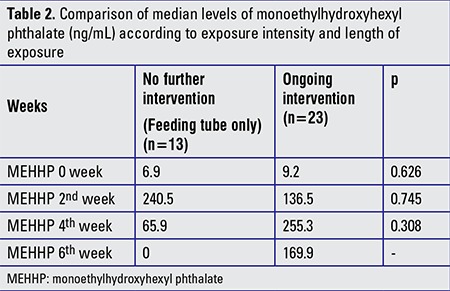
Comparison of median levels of monoethylhydroxyhexyl phthalate (ng/mL) according to exposure intensity and length of exposure

**Table 3:**
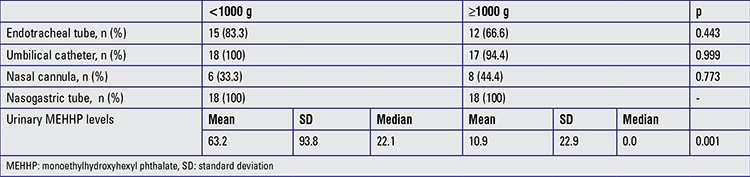
Comparison of exposure intensity and urinary monoethylhydroxyhexyl phthalate levels (ng/mL) of patients on admission to the neonatal intensive care units (the first 3 days) according to birth weight category

## References

[1] (2002). ATSDR. Agency for Toxic Substances and Disease Registry.

[2] Jaakkola JJ, Knight TL (2008). The role of exposure to phthalates from polyvinyl chloride products in the development of asthma and allergies: a systematic review and meta-analysis. Environ Health Perspect.

[3] Bornehag CG, Sundell J, Weschler CJ, Sigsgaard T, Lundgren B, Hasselgren M, Hägerhed-Engman L (2004). The association between asthma and allergic symptoms in children and phthalates in house dust: a nested case-control study. Environ Health Perspect.

[4] Loff S, Kabs F, Subotic U, Schaible T, Reinecke F, Langbein M (2002). Kinetics of diethylhexyl-phthalate extraction from polyvinylchloride-infusion lines. J Parenter Enteral Nutr.

[5] NTP-CERHR 2005 Expert Panel Updated on the Reproductive and Developmental Toxicity of Di(2-ethylhexyl) Phthalate. Research Triangle Park, NC: National Toxicology Program, Center for the Evaluation of Risks to Human Reproduction.

[6] Peck CC, Albro PW (1982). Toxic potential of the plasticizer Di(2-ethylhexyl) phthalate in the context of its disposition and metabolism in primates and man. Environ Health Perspect.

[7] Latini G, Del Vecchio A, Massaro M, Verrotti A, DE Felice C (2006). In utero exposure to phthalates and fetal development. Curr Med Chem.

[8] Main KM, Mortensen GK, Kaleva MM, Boisen KA, Damgaard IN, Chellakooty M, Schmidt IM, Suomi AM, Virtanen HE, Petersen DV, Andersson AM, Toppari J, Skakkebaek NE (2006). Human breast milk contamination with phthalates and alterations of endogenous reproductive hormones in infants three months of age. Environ Health Perspect.

[9] (2000). National Toxicology Program, Center for the Evaluation of Risks to Human Reproduction. NTP-CERHR Expert Panel Report on Di-isononyl Phthalate. Alexandria, VA: Center for the Evaluation of Risks to Human Reproduction, US Department of Health and Human Services.

[10] (1982). National Toxicology Program. Carcinogenesis Bioassay of Di-(2-ethylhexyl) Phthalate (CAS No. 117–81-7) in F344 Rats and B6C3F1 Mice (Feed Study). Research Triangle Park, NC.

[11] National Toxicology Program, Center for the Evaluation of Risks to Human Reproduction. Final CERHR expert panel reports on 7 phthalate esters.

[12] Blount BC, Silva MJ, Caudill SP, Needham LL, Pirkle JL, Sampson EJ, Lucier GW, Jackson RJ, Brock JW (2000). Levels of seven urinary phthalate metabolites in a human reference population. Environ Health Perspect.

[13] Barr DB, Silva MJ, Kato K, Reidy JA, Malek NA, Hurtz D, Sadowski M, Needham LL, Calafat AM (2003). Assessing human exposure to phthalates using monoesters and their oxidized metabolites as biomarkers. Environ Health Perspect.

[14] Shea KM, American Academy of Pediatrics Committee on Environmental Health (2003). Pediatric exposure and potential toxicity of phthalate plasticizers. Pediatrics.

[15] Leeder JS, Kearns GL (1997). Pharmacogenetics in pediatrics: Implications for practice. Pediatr Clin North Am.

[16] Calafat AM, Needham LL, Silva MJ, Lambert G (2004). Exposure to di-(2-ethylhexyl) phthalate among premature neonates in a neonatal intensive care unit. Pediatrics.

[17] Green R, Hauser R, Calafat AM, Weuve J, Schettler T, Ringer S, Huttner K, Hu H (2005). Use of di (2-ethylhexyl) phthalate-containing medical products and urinary levels of mono (2-ethylhexyl) phthalate in neonatal intensive care unit infants. Environ Health Perspect.

[18] Weuve J, Sanchez BN, Calafat AM, Schettler T, Green RA, Hu H, Hauser R (2006). Exposure to phthalates in neonatal intensive care unit infants: urinary concentrations of monoesters and oxidative metabolites. Environ Health Perspect.

[19] Kato K, Silva MJ, Reidy JA, Hurtz D, Malek NA, Needham LL, Nakazawa H, Barr DB, Calafat AM (2004). Mono(2-ethyl-5-hydroxyhexyl) phthalate and mono-(2-ethyl-5-oxohexyl) phthalate as biomarkers for human exposure assessment to di-(2-ethylhexyl) phthalate. Environ Health Perspect.

[20] Kato K, Silva MJ, Brock JW, Reidy JA, Malek NA, Hodge CC, Nakazawa H, Needham LL, Barr DB (2003). Quantitative detection of nine phthalate metabolites in human serum using reversed-phase high-performance liquid chromatography-electrospray ionization-tandem mass spectrometry. J Anal Toxicol.

[21] Silva MJ, Malek NA, Hodge CC, Reidy JA, Kato K, Barr DB, Needham LL, Brock JW (2003). Improved quantitative detection of 11 urinary phthalate metabolites in humans using liquid chromatography-atmospheric pressure chemical ionization tandem mass spectrometry. J Chromatogr B Analyt Technol Biomed Life Sci.

[22] Takatori S, Okamoto Y, Kitagawa Y, Hori S, Izumi S, Makino T, Nakazawa H (2008). Simulated neonatal exposure to DEHP and MEHP from PVC enteral nutrition products. Int J Pharm.

[23] Chiellini F, Ferri M, Latini G (2011). Physical-chemical assessment of di-(2-ethylhexyl)-phthalate leakage from poly(vinyl chloride) endotracheal tubes after application in high risk newborns. Int J Pharm.

[24] Latini G, Avery GB (1999). Materials degradation in endotracheal tubes: a potential contributor to bronchopulmonary disease. Acta Paeditr.

[25] Zhang YH, Lin L, Cao Y, Chen B, Zheng L, Ge RS (2009). Phthalate levels and low birth weight: a nested case-control study of Chinese newborns. J Pediatr.

